# Robust Effects of Graphene Oxide on Polyurethane/Tourmaline Nanocomposite Fiber

**DOI:** 10.3390/polym13010016

**Published:** 2020-12-23

**Authors:** Yuanchi Zhang, Jinlian Hu

**Affiliations:** 1 Centre for Translational Medicine Research & Development, Shenzhen Institutes of Advanced Technology, Chinese Academy of Sciences, Shenzhen 518055, China; zhangyc@siat.ac.cn; 2 Department of Biomedical Engineering, City University of Hong Kong, Kowloon, Hong Kong 999077, China

**Keywords:** graphene oxide, tourmaline, polyurethane fiber, negative ions, robust effects

## Abstract

The use of energy therapy including tourmaline/negative ions has gained huge popularity due to their long-standing historical evidence in improving human health and the technology development. However, the limitations of tourmaline based polyurethane fibers including the unsatisfied mechanical properties and negative ions releasing performances hind their further applications for wearable energy therapy. In this study, graphene oxide was modified within the polyurethane/tourmaline nanocomposite and then the wet-spinning method was used to prepare the fibers. As expected, the results proved that polyurethane/tourmaline/graphene oxide fiber had enhanced Young’s modulus (8.4 MPa) and tensile stain at break (335%). In addition, the number of released negative ions from polyurethane/tourmaline/graphene oxide fiber was significantly improved 17 times and 1.6 times more than that of neat polyurethane fiber and polyurethane/tourmaline fiber, respectively. Moreover, the releasing number of negative ions was significantly decreased after being applying voltage. We envision that the proposed polyurethane/tourmaline/graphene oxide fiber will provide valuable insights into the development of the wearable energy products.

## 1. Introduction

With intensive awareness in healthcare and life quality in developed parts of the world, a mineral-based wearable therapy fashion is flourishing as clothing, jewelry, accessories and devices since new technologies have allowed us to utilize a variety of natural materials and their composites for attractive wearable products [1,2,3,4]. Negative ions are the most renowned source from nature minerals with the therapeutic effect, which usually refers to those small ions with an extra electron attached such as O_2_^−^(H_2_O)_n_ and OH^−^(H_2_O)_n_ [5,6]. They are created by various effects on water and oxygen in air by thunderstorm, waves, evaporation and minerals, which can be found in different environment with different concentrations. They are also called “air vitamins” with functions including the antibacterial ability, sterilization and healthcare benefits, having a very important influence on the life activities of human and other organisms [1,7,8]. Their therapeutic abilities can neutralize free radicals in body and help to purify blood, promote blood circulation, stimulate cell metabolism and reduce cell damage [9,10]. Due to the massive advantages, materials releasing negative ions and methods improving negative ions have been always sought after.

Tourmaline is a crystalline boron silicate mineral compounded with elements such as aluminum, iron, magnesium, sodium, lithium or potassium. Its general formula is widely accepted as XY_3_Z_6_(BO_3_)_3_Si_6_O_18_V_3_W, where X is Na, Ca, K or vacancies; Y is Li, Mg, Al, Fe^2^^+^, Fe^3^^+^, Mn^2^^+^, Cr^3^+, Ti^4^^+^, Zn or vacancies; Z is the cationic element like Mg, Al, Fe^3^^+^, Cr^3^+ and V^3^^+^ and W is the anionic element like OH^−^, F^−^ or O^2^^−^ [11,12]. Tourmaline presents its piezoelectric and pyroelectric characteristics upon the temperature or pressure conditions change, so it can form the electric potential difference and exhibit spontaneous surface electric fields [13,14]. With such specific property, tourmaline crystal could ionize the surrounding water and oxygen molecules to generate negative ions spontaneously and permanently. Tourmaline has been widely applied for healthcare products [15], air or water purification [16] due to the negative air releasing functions. More importantly, it is always expected that the tourmaline particles could be loaded well in polymeric fibers, thus the functional and healthy wearable products can be further expanded [9,17,18]. For instance, Leonard D. Tijing et al. prepared an electrospun nanocomposite fiber made of polyurethane (PU)/tourmaline nanoparticles (TNs) and demonstrated its antibacterial performance [13].

PU is a kind of thermoplastic polymers, which the advantages including good mechanical and thermal properties, satisfied abrasion resistance and fatigue life and water insolubility provide PU an increasing popularity in worldwide researchers [19,20,21,22,23,24]. In general, PU fibers have the excellent elasticity and extensibility, which can be used for making elastic textile products such as sportswear, corduroy fabric, medical bandage and surgical suture. The next-to-skin garments made of PU fibers can contribute to comfortable and flexible feelings. In addition, the elasticity and extensibility of PU fiber can satisfy the requirement of various body motions and reduce the limitation feelings of body. From the point of negative ion releasing, due to the interaction between fiber and the human body, the excellent elasticity and extensibility will be beneficial to higher releasing negative ions. In spite of many benefits of PU and TN, however, some concerns still exist on mechanical properties of the TN-based composite fibers. Due to the different surface properties between TN and the polymer matrix, poor dispersion of TN often results in decreased properties of the composite, thus affect the overall performances including the negative ions releasing [13]. Previously, our group found that mineral nanoparticles like hydroxyapatite in the physical blending PU composites could have an aggregation to decrease the modulus of the composite [22]. Good news is the negative effects could be improved through forming a stable molecular construction and we found graphene oxide (GO) is an effective tool to consolidate the PU composite [22,25]. It is well known that graphene composed of sp^2^-bonded carbon atoms has excellent mechanical, optical, conductive properties and good biocompatibility due to its special structure [26,27]. Similarly, GO possess the same structure of single planar sheet while has more oxygen containing groups on the surface, which is often considered as the fillers to modify the materials. Researchers have proved that GO could enhance effectively the mechanical properties of the nanocomposites and its good biocompatibility also provides huge potentials in biomedical applications [28]. For instance, Pokharel et al. reported that 3 wt % GO based PU composite had a 280.5% increase in modulus compared that of pristine PU [29]. Additionally, it is expected that the electrostatic repulsion between GO sheets could have an interaction with the TN due to its piezoelectric and pyroelectric properties, resulting in a homogeneous dispersion in the PU nanocomposite to be applied easily for wearable products [30]. In fact, researchers have reported that graphene materials could promote negative ions releasing performances [31]. Additionally, a pervious study reported the preparation of the TN/GO nanocomposite and their good purification capacity for removing organic dye and heavy metal [32].

**Figure 1:**
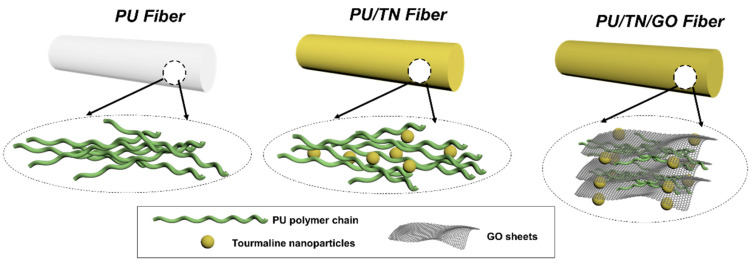
Schematic diagram of the PU and its nanocomposite fibers.

Herein, we took advantages of the GO nanofiller to develop a multi-modified PU/TN/GO nanocomposite fiber. The main objective of this study is to achieve a robust performance including the improved mechanical and negative ions releasing properties. In this study, the TN and GO were physically blended with the PU matrix one by one. Then, the pristine PU and its composites including PU/TN and PU/TN/GO were fabricated to fibers through a simple wet-spinning process (Figure 1). As a proof of concept, Fourier transform infrared (FTIR) spectroscopy, scanning electron microscopy (SEM) and energy dispersive spectroscopy (EDS) were conducted to demonstrate the successful modifications and the structures of the composites. In addition, differential scanning calorimetry (DSC) and tensile tests were carried out to confirm the thermal and mechanical performances. Moreover, to investigate the effects of electricity on the ionizing ability of TNs, negative ions releasing performances of the samples with and without applied voltage were measured by a negative ions’ tester.

## 2. Materials and Methods

### 2.1. Materials

PU was prepared according to our pervious study [33]. Briefly, dried polyethylene glycol (PEG, Mn = 2000 g/mol, Sigma-Aldrich Co. Ltd., Shanghai, China), polypropylene glycol (PPG, Mn = 600 g/mol, Sigma-Aldrich Co. Ltd., Shanghai, China), 4,4-diphenylmethane diisocyanate (MDI, Sigma-Aldrich Co. Ltd., Shanghai, China) and dimethylacetamide (DMAc) were mixed with mechanically stirring at 80 °C for 2 h. Then, 2,2-Bis(hydroxymethyl)propionic acid (DMPA, Sigma-Aldrich Co. Ltd., Shanghai, China), 1,2-diaminoethane (EN, Aladdin Chemistry Co. Ltd., Shanghai, China) and MDI were successively added under the same temperature and mixed for another 2 h, where the carboxylic acid group of the DMPA was neutralized by the triethylamine at 50 °C for 2 h. Finally, the solution was pour into the PTFE model at 80 °C for 24 h to obtain the PU solid. TNs were obtained from the Sharpwell Technology Ltd. (HongKong, China). GO, dimethylacetamide (DMAc) sheets were obtained from Sigma-Aldrich (Shanghai, China).

### 2.2. Preparation of PU/TN and PU/TN/GO

**Figure 2:**
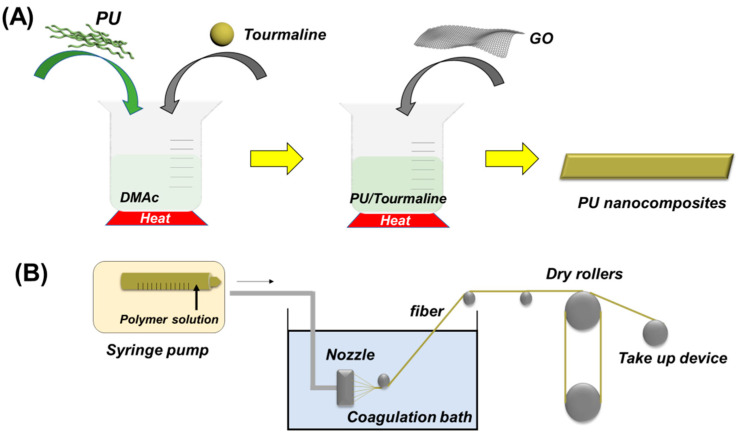
Preparation of the PU nanocomposite fibers: (**A**) preparation of the PU nanocomposite and (**B**) wet spinning process.

At first, as shown in Figure 2A, 20 wt % TNs (relative to the total weight) and PU were added and then mixed in certain DMAc (the concentration of total samples was 15 wt %) with stirring at 70 °C for 2 h, followed by ultrasonic dispersion for 1 h. Until the homogenous solution was obtained, TPU/TN/DMAc solution were poured into specific mold for being dried in the oven at 80 °C over night to get the final film product. As for PU/TN/GO, 2 wt % GO sheets (relative to the total weight) were added in the PU/TN/DMAc solution with mechanically stirring at 70 °C for 2 h, also followed by ultrasonic dispersion for 1 h. After that, PU/TN/GO film can be attained after being heated at 80 °C in the oven for 24 h during which all DMAc was evaporated.

### 2.3. Preparation of Wet Spinning Fibers

Nanocomposite fibers were prepared by the wet spinning method using a wet spinning instrument for lab use. For preparing the spinning solution, the samples including PU, PU/TN and PU/TN/GO were dissolved in DMAc with stirring at 70 °C for 2 h. The weight ratio of the samples was 15%. After that, the uniform solution was placed into the needle. As illustrated in Figure 2B, the spinning solution was extruded uniformly to the spinneret with the speed of 1 mL/min. Fibers were formed by solidification in a coagulation bath in water at room temperature. The drawing process was performed under a drawing ratio at 50 rpm. Finally, the drawn fibers were transferred to the heating chamber at 50 °C for 24 h to remove solvents and outside water.

### 2.4. Characterization of Wet Spinning Fibers

FTIR spectra of samples were used to confirm the modifications of TN and GO with PU by using a 2000 FTIR spectrometer (Perkin–Elmer, Suzhou, China) with attenuated-total-reflectance accessories in the wavenumber range of 500–4000 cm^−1^ at room temperature. Each spectrum was obtained by averaging 16 scans with a 4 cm^−1^ resolution. Surface morphologies of the fibers were recognized by the SEM (FEI Inspect F50, Thermo Fisher, Hong Kong, China) at an accelerating voltage of 5 kV. Elemental analysis of the selected area in fibers was obtained using an EDS attached to SEM. The images were captured by a digital single-lens reflex camera (Canon, EOS 600D, Hong Kong, China).

Differential scanning calorimetry (DSC, Perkin Elmer DSC7, Shanghai, China) was utilized to measure the glass transition temperatures (*T*_g_) of fibers. At first, the samples were heated to 100 °C and then cooled to 20 °C at a rate of 10 °C min^−1^. After remaining at this temperature for 5 min, a second scan was processed from 20 to 100 °C at the same rate, from which data were used for analyzing. The INSTRON 5566 tester (Hong Kong, China) was used to conduct the tensile tests (refer to ASTM C1557-20) of the fibers at room temperature (RT) with an extension rate of 5 mm min^−1^. Tensile strength, tensile strain at break, Young’s modulus and maximum load at break were derived from the stress–strain curves to compare the mechanical properties of various fibers. Negative ions releasing performance of fibers with and without voltage were performed by the negative ions tester (com-3010 Pro, Tokyo, Japan). The weight of each sample was 0.5 g and the voltage was 10 V given by a DC power supply (Hong Sheng, Shenzhen, China).

## 3. Results and Discussion

### 3.1. Structures and Morphologies of PU and Its Nanocomposites Fibers

**Figure 3:**
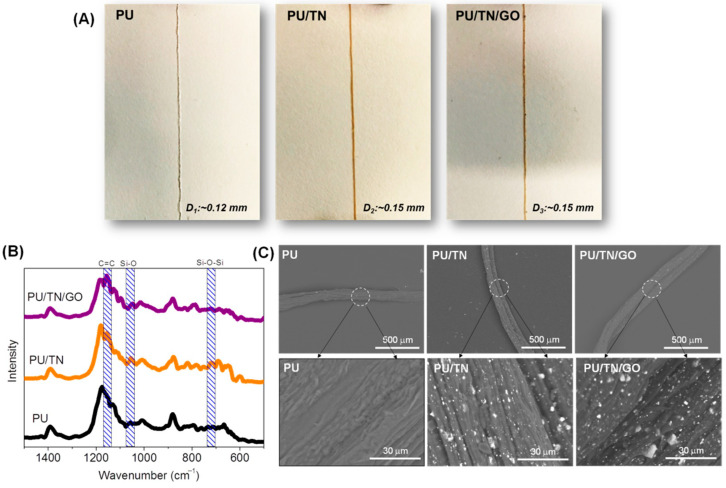
Structure and morphologies of the PU and its nanocomposite fibers: (**A**) images; D_1_: diameter of PU fiber; D_2_: diameter of PU/TN fiber and D_3_: diameter of PU/TN/GO fiber; (**B**) magnified FTIR spectra and (**C**) SEM images; white line area and arrows: magnified area.

The images of PU, PU/TN and PU/TN/GO fibers are shown in the Figure 3A. Pristine PU had a diameter of 0.12 mm and was a white color. After being blended with TN, the diameter of PU/TN was about 0.15 mm and the color became brown due to the existence of TN. As regarding GO, black particles could be found in the image of PU/TN/GO fiber that its diameter was also 0.15 mm. The increase in diameters of the composite fibers may be attributed by the introduction of nanofillers under the same fiber collection speed, which increased the viscosity of the spinning solution [34]. Figure 3B shows the magnified FTIR spectra of PU and its nanocomposite fibers. Compared with the spectrum of pristine PU, the spectrum of PU/TN presented two characteristic bands: 1048 cm^−1^ (Si–O) and 719 cm^−1^ (Si–O–Si), which indicated the existence of TN [35]. In addition, compared with pristine PU, the typical band representing C=C (1157 cm^−1^) in the spectrum of PU/TN/GO was enhanced, which suggested the modification of GO in the nanocomposite. Figure 3C is the SEM images of various fibers. Pristine PU showed a rough surface that being consistent with previous references [25]. By comparison, white particles could be found in the surface of the PU/TN fiber. In addition, both particles and sheets could be observed on the surface of PU/TN/GO. After introducing GO sheets, the TNs could be found dispersed better on the surface, which may be contributed by the strong electrostatic repulsion between TNs and GO due to the negatively charged groups on GO sheets [30]. These results suggested the TN and GO were successfully introduced in the nanocomposite fibers.

**Figure 4:**
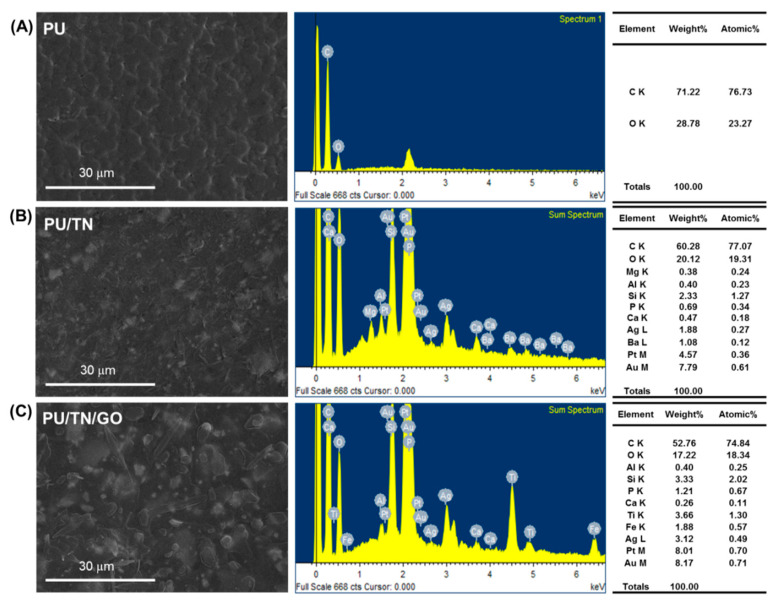
SEM images of cross sections and corresponding EDS peaks of (**A**) PU; (**B**) PU/TN and (**C**) PU/TN/GO.

Figure 4 presents the SEM image and EDS spectra of PU and its nanocomposite fibers’ cross section. The morphology features were similar with that in Figure 3C. After introducing the GO sheets, the TNs could be dispersed well in PU. Compared with neat PU, EDS spectrum of PU/TN showed strong peaks of elements including Ca, Mg, Al, Si, P, Ca, Ag, Ba, Pt and Au besides C and O (Figure 4B), which were typical TN elements [35,36]. Furthermore, after introducing GO, a difference could be found between the EDS spectra of PU/TN and PU/TN/GO. The strong peaks of elements like Mg and Ba decreased even disappeared while some elements increased like Ti and Fe. This may be attributed to the different scan period. Another cause might be the effects of GO’s single planar sheets structure. Some publications reported that graphene materials had a shielding effect on radiation [37], which will be investigated in our further study. The EDS investigation confirmed the presence of TN and GO in the nanocomposite fibers.

### 3.2. Thermal and Mechanical Properties of PU and Its Nanocomposites Fibers

**Figure 5:**
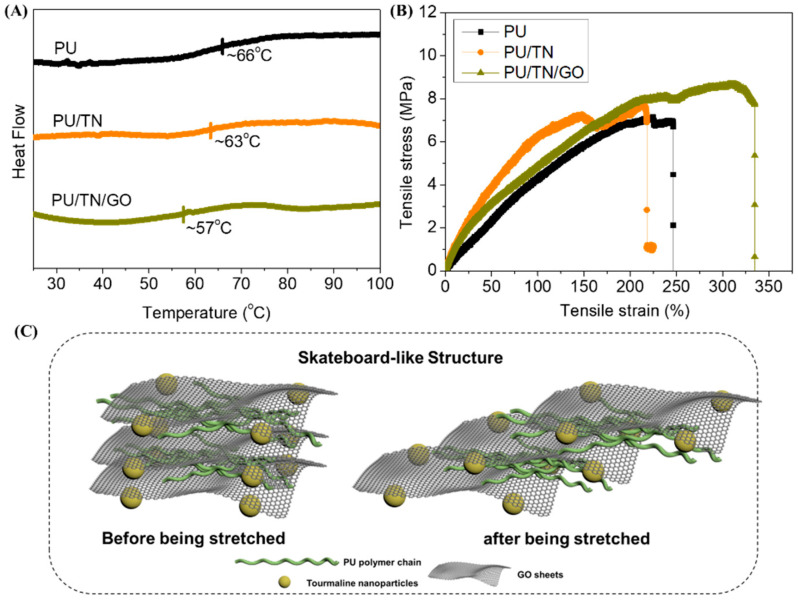
Thermal (**A**) and mechanical properties (**B**) of PU and its nanocomposite fibers and (**C**) skateboard-like structure of PU/TN/GO before and after being stretched.

*T*_g_ of PU, PU/TN and PU/TN/GO was investigated by DSC, as shown in Figure 5A. Compared with neat PU, *T*_g_ of PU/TN decreased to 63 °C. Based on published reports, *T*_g_ could represent the mobility of the soft segment (polymer chains) in the matrix [25,36]. In this case, after adding TN, dispersed nanoparticles might reduce molecular interaction among polymer chains leading to an increase of mobility. In addition, after introducing GO, *T*_g_ was further decreased to 57 °C. According to the literature, GO based PU composite could form an intercalated structure [25]. Besides, TN could weaken the electrostatic repulsion between GO sheets due to its electrostatic field effect [30]. So, the nanoparticles and single planar GO sheets might form a skateboard-like structure (Figure 5C) that could further increase the mobility of polymer chains to decrease the *T*_g_ value.

What is interesting is the results of the mechanical tests on those fibers. The data including tensile strength, tensile strain at break, Young’s modulus and maximum load at break derived from the stress–strain curves (Figure 5B) were shown in Table 1. Although the stain at break of PU/TN (215%) had a little decrease compared to that of neat PU (247%), Young’s modulus of PU/TN (8.3 MPa) had 200% improvement with the reference of its pristine PU (4.3 MPa). The stain at a break of PU/TN (215%) had a little decrease compared to that of neat PU (247%). This might be ascribed to the negative impacts of nanoparticles’ aggregation, which disturbed the polymer flexibility partly in the elongated phase leading to the stress concentration of the composite fiber [25,38]. In addition, Young’s modulus of PU/TN (8.3 MPa) had 200% improvement with the reference of its pristine PU (4.3 MPa). This should be caused by the enhanced effect of the TNs and the metal affinity interaction between the positively charged metal ions of TNs and the negatively charged oxygen atoms of PU [22]. As for PU/TN/GO, as described previously, besides the high modulus, GO also assisted to form a stable structure in the nanocomposites. Therefore, the Young’s modulus (8.4 MPa) and the stain at break (335%) even the tensile strength (8.7 MPa) of PU/TN/GO had a coalesced improvement. In contrast, some researchers reported in certain polymer/nanofiller systems, mechanical performances could be decreased with the increasing of the nanofillers [39]. In this proposed system, the introduction of GO could maintain the strength and meantime have an improved stretchability, which is beneficial for the further applications of tourmaline-based textiles. These results also demonstrated the robust effects of GO in this nanocomposite system.

### 3.3. Negative Ions Releasing Performances

**Figure 6:**
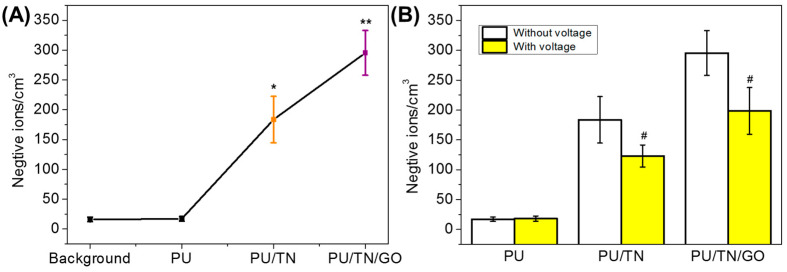
Negative ions releasing performances of PU and its nanocomposite fibers: (**A**) in the common environment and (**B**) the effect of voltage on negative ions releasing; *, significant difference compared to neat PU; **, significant difference compared to PU/TN and #, significant difference compared to itself without voltage, *p* < 0.05.

The number of negative ions was one of the important properties of tourmaline-based textiles/products. Negative ions releasing number from various samples was measured and the number of negative ions in the background (the same environment without samples) was regarded as the control group. The results were shown in Figure 6A. The negative ions released from the background and neat PU were few (16/cm^3^ and 17/cm^3^), which also confirmed the results in published reports. As for PU/TN nanocomposite, the negative ions releasing number was significantly increased (184/cm^3^), which was near 11 times more than that of neat PU. In addition, after introduction of GO, released negative ions from PU/TN/GO were further improved (296/cm^3^), which was about 17 times as much as that released from neat PU. More importantly, the significant difference of releasing number could be found between PU/TN and PU/TN/GO nanocomposites. The negative ions from PU/TN/GO were around 1.6 times more than that from PU/TN. Previous studies have found that graphene materials could improve the releasing of negative ions in the nanocomposite through enhancing the spontaneous polarization effect of tourmaline [40]. In this work, the results also proved GO could improve the negative ions releasing performance of the PU nanocomposite. TN has piezoelectric and pyroelectric characteristics to ionize surrounding air to generate negative ions. Therefore, to investigate the effect of electricity on negative ions releasing performances of the nanocomposites, the number of negative ions was tested after applying 10 V voltage to the samples. The results were shown in the Figure 6B. For neat PU, the voltage had little effects on it due to few negative ions released. As regards PU/TN and PU/TN/GO nanocomposites, after being applied voltage, the number of negative ions released from both samples was significantly decreased (123/cm^3^ and 198/cm^3^, respectively). This might be because after applying voltage, the ionizing ability of TNs was reduced. In addition, some positive static charges might generate with applied voltage, then be neutralized with the released negative ions. As a result, the measured negative ions decreased. The effects and the mechanism might be useful for developing an electrostatic eliminator in textile products, which will be further studied in our future work.

## 4. Conclusions

This work developed a GO-modified PU/TN nanocomposite fiber through a wet-spinning approach with enhanced mechanical properties and negative ions releasing performance. Compared with pristine PU fiber, PU/TN nanocomposite fiber had significant improvement in the Young’s modulus (200%) and released negative ions (>1000%). In addition, the experimental results demonstrated that after adding GO, mechanical properties including tensile strength, tensile strain and maximum load at break of the PU/TN/GO fiber were further improved referring to that of the PU/TN fiber. These could be attributed to the benefits from the interaction of GO and TN and the stable “skateboard-like” structure. What is more, the modification of GO was also demonstrated to significantly promote negative ions releasing performance compared with PU/TN fiber (160% improvement). After applying voltage, the measured number of negative ions released from both nanocomposite fibers had a significant decrease, which might be caused by the reduced ionization of TN, and neutralization between negative ions and positive static charges. We believe that this study has potential for the further development and application of the negative ions textiles and wearable energy therapy in the future.

## Figures and Tables

**Table 1:** Mechanical properties of PU and its nanocomposite fibers.

Items	PU	PU/TN	PU/TN/GO
Tensile strength (MPa)	7.0	7.7	8.7
Tensile strain at break (%)	247	215	335
Young’s modulus (MPa)	4.3	8.3	8.4
Maximum Load at break (N)	0.08	0.05	0.10
